# Ultra-Pulsed CO_2_ Laser Osteotomy: A New Method for the Bone Preparation of Total Knee Arthroplasty

**DOI:** 10.3389/fbioe.2022.858862

**Published:** 2022-04-29

**Authors:** Tianfei Ran, Chuanchuan Lin, Tianying Ma, Yinyin Qin, Jie Li, Yuan Zhang, Yuan Xu, Changqing Li, Min Wang

**Affiliations:** ^1^ Department of Orthopedics, Xinqiao Hospital, Third Military Medical University, Chongqing, China; ^2^ Department of Blood Transfusion, Xinqiao Hospital, Amy Medical University (Third Military Medical University), Chongqing, China

**Keywords:** CO_2_ laser osteotomy, total knee arthroplasty, cementless, thermal damage, bone preparation, adhesion

## Abstract

Cementless total knee arthroplasty (TKA) can achieve long-term biological fixation, but its application is limited by the risk of early aseptic loosening. One of the important reasons for early aseptic loosening is that mechanical osteotomy tools cannot achieve ideal bone preparation because of poor accuracy and serious bone tissue damage produced by them. Therefore, we designed an ultra-pulsed CO_2_ laser osteotomy system to solve these problems. To reveal the safety at the tissue and cell levels of the ultra-pulsed CO_2_ laser osteotomy system, a series of experiments on distal femur osteotomy in animals were performed. Then, the bone surface characteristics were analyzed through scanning electron microscopy, and the bone thermal and mechanical damage was evaluated *via* histological analysis. Finally, mesenchymal stem cells (MSCs) were inoculated on the bone surfaces prepared by the two osteotomy tools, and the effect of cell adhesion was analyzed through a confocal laser scanning microscope (CLSM). We successfully achieved TKA bone preparation of animal knees with the ultra-pulsed CO_2_ laser osteotomy system. Moreover, the biological evaluation results indicated that compared with the traditional mechanical saw, the laser can preserve the natural bone structure and cause no thermal damage to the bone. In addition, CLSM examination results showed that the laser-cut bone surface was more conducive to cell adhesion and infiltration than the bone surface cut by a mechanical saw. Overall, these results indicate that ultra-pulsed CO_2_ laser can achieve non-invasive bone cutting, which can be a new option for TKA bone preparation and has the potential to lead in the future.

## Introduction

Total knee arthroplasty (TKA) is the most effective treatment for degenerative diseases of the knee (Lotke et al., 1976; Katz et al., 2021). In TKA, cemented or cementless implants or a hybrid of the two is fixed to the bone surface. The cemented fixation can achieve rapid stability between the implants and bone surface but cannot achieve the integration between the bone and implants, raising concerns about the long-term durability of cemented fixation (Miller et al., 2013). Theoretically, the advantage of cementless biological implants is that it can achieve permanent biological fixation between bone and implants (Dalury, 2016; Behery et al., 2017). However, limited application of cementless TKA has been reported in studies (Parker et al., 2001; Nakama et al., 2012; List et al., 2017), and defects in traditional bone osteotomy methods are one of the key reasons.

We believe that the defects of traditional bone osteotomy lie in the following aspects: first, it is difficult to achieve accurate bone cutting with traditional mechanical osteotomy tools (Macdonald et al., 2004). The inevitable shaking perpendicular to the osteotomy plane during mechanical cutting will eventually increase the gap and micro motion at the junction between the implant and bone surface (Kamath et al., 2021), which may lead to prosthesis failure. Second, the preparation of dense sclerotic bone can cause the saw blade to skive, and this adversely affects flush surface cutting. In addition, the sclerotic bone may further generate excessive heat during mechanical osteotomy, which may lead to thermal necrosis (Eriksson et al., 1984). Third, the saw will also cause mechanical damage to the bone, destroy the microstructure of bone tissue, and produce a significant amount of bone debris. This debris is bone sequestra, a cause of aseptic necrosis, which will then retard bone regeneration by increasing the time for the macrophages to cleanse the wound (Giraud et al., 1991). Therefore, it is important to find a new way of osteotomy in TKA.

Recently, laser, as the most promising technology to replace traditional mechanical osteotomy tools such as electric saws and drills to achieve bone cutting, has attracted the attention of researchers (Baek et al., 2015). Laser has unique advantages such as non-contact, non-vibration (Berrocal et al., 2007), high-precision, and arbitrary-geometry cutting. This is especially true when the laser beam with a diameter of only micron order is combined with advanced computer control, and it can cut bones more accurately (Baek et al., 2015; Marcello et al., 2018).

Theoretically, the advantages of laser can perfectly suit the needs of TKA bone preparation. However, the thermal damage of laser has always puzzled researchers (Gunaratne et al., 2016). With the improvement in laser design, the heat has been effectively controlled. To test that laser osteotomy can achieve ideal TKA bone preparation, we designed a new ultra-pulsed CO_2_ laser osteotomy system. Then, this system and the traditional mechanical saw were used to perform distal femur osteotomy in animals for comparison. We analyzed the osteotomy surface structure, evaluated the thermal effect on bone and bone cells, and studied the biological characteristics of bone surface to reveal the safety of the ultra-pulsed CO_2_ laser osteotomy system.

## Materials and Methods

### Materials

4% paraformaldehyde, Triton X-10, and 10% EDTA solution were provided by Biosharp (Anhui, China). The Hematoxylin–Eosin (HE) Staining Kit and DAPI were purchased from Beyotime (Chongqing, China). A Calcein/PI Live/Dead Cell Viability Kit was supplied by Sigma Chemical Co. (St. Louis, MO, United States). Rhodamine phalloidin, Alexa Fluor 488, and anti-paxillin antibody were supplied by Abcam (Shanghai). Sprague–Dawley (SD) rats aged 12 weeks (400 ± 40 g) were provided by Hunan SJA Laboratory Animal Co.. LTD. (Hunan, China).

### Laser System and Animal TKA Osteotomy

**FIGURE 1:**
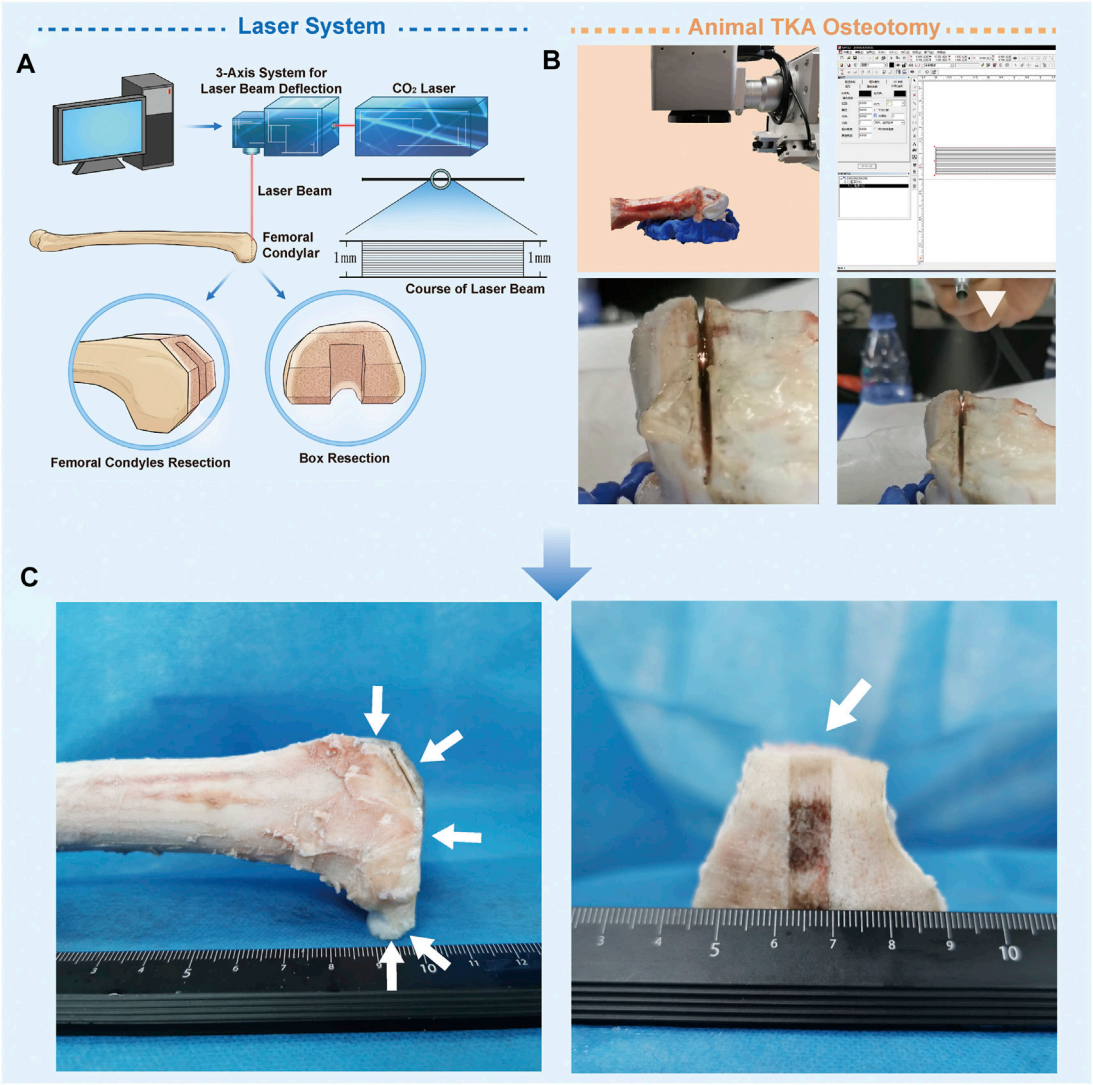
Ultra-pulsed CO_2_ laser osteotomy of the distal femur of sheep. **(A)** Schematic chart of the ultra-pulsed laser CO_2_ laser osteotomy system **(B)** Operational process of the sheep distal femur bone-cutting surface in total knee replacement using this system; the white triangle indicates the self-made water spray device **(C)** Distal femur after laser cutting.

The parameters of the ultra-pulsed CO_2_ laser osteotomy system (Shenzhen KBF Laser Technology Co., Ltd., China) are shown in Table 1. In this study, the pulse duration is 25 µs, the single pulse energy is 25 mJ, and the focal spot diameter after the beam passes through the galvanometer is 60 μm. The galvanometer (AxialScAN-20-30, Germany) can dynamically control the working position of the laser focus on X, Y, and Z-axes through the software Marking Mate 3D (Version 2.7). All cutting processes in this study are performed by line filling cutting, with a line width of 1 mm (the course of the laser beam is shown in Figure 1A). After completing a scanning filling, the galvanometer controls the focus to drop 0.05 mm along the *Z*-axis, and the cutting speed is 80 mm/s. The model of the laser osteotomy system is shown in Figure 1.

**TABLE 1:** Parameters of the ultra-pulsed CO_2_ laser osteotomy system.

Parameters	
Wavelength	9.3 μm
Average power	5 W
Peak power	500 W
Beam waist diameter	2.2 mm
Full divergence angle	6.2 mrad
Rise time	<10 μs
Fall time	<15 μs
Pulse repetition frequency	500 Hz

We used this system to perform TKA osteotomy on the isolated knee of sheep aged one year and weighing 40 kg (the shape of the femoral condyle in the sheep is similar to that in humans). The experiments were performed in accordance with the protocols approved by the Ethics Committee of TMMU (Approval Number: 20170002). Before cutting, the femur was fixed on the super-light clay, which could be shaped quickly. Then, laser osteotomy was performed in accordance with the TKA osteotomy method. After each resection, the position of the femur was manually adjusted. The distal femur, anterior cortex, anterior bevel, posterior condyle, and posterior bevel were sequentially sectioned, and finally, box resection was performed. The whole process was sprayed with a self-made water sprayer, 1 ml/min (Kuttenberger et al., 2008). Water acts as a coolant to reduce thermal injury, and the micro explosion of water irradiated by the laser can accelerate cutting (Zhang et al., 2012). The distal femur after laser cutting is shown in Figure 1C.

### Surface Characterization and Thermal Analysis

#### Specimen and Grouping

For animal protection and subsequent bioactivity analysis of the osteotomy surface, the experimental animal model was changed to an SD rat. The specimens were the femurs of rats dissected immediately after cardiac injection of excessive anesthesia. The experimental group was the laser osteotomy group (L group), and the control group was the mechanical saw group (S group).

##### Femoral Osteotomy

Osteotomy was performed at the distal femur to analyze the cancellous bone and performed in the middle of the femur to analyze the cortical bone. In the L group, the aforementioned laser system was used for cutting. Briefly, after fixing the femur, it was completely resected at 5 mm from the distal femur to obtain the cancellous bone specimen, and then, the femur was cut in the middle section to obtain the cortical bone specimen. A water spray (1 ml/min) was used during cutting. In the S group, a mechanical saw (saw blade thickness 1mm, UNIVERSAL, American) was used to cut the femur at the same site. To minimize interference, each cut was performed at a maximum round-trip frequency with water spraying (1 ml/min). Finally, the specimens were collected. There were 18 samples of cancellous bone and 18 samples of cortical bone from 18 rats, half of which were used for surface characterization and half for histological analysis.

### Surface Characterization.

The specimens were gently rinsed with normal saline at room temperature and then freeze-dried. Briefly, the samples were sequentially refrigerated at 4°C for 30 min, −20°C for 12 h, and −80°C for 7 days. The freeze-drying apparatus (FD-1A-50, BiLon, Beijing) was used to prepare the samples for 24 h. After spraying gold, the morphology of the cutting surface was observed by scanning electron microscopy (SEM) (S-3400N, HITACHI, Japan). The surface roughness of the samples was examined by using a 3D laser confocal scanning microscope (OLS5000, Olympus, Japan), and the results were analyzed by multifile analyzer software. The surface roughness was measured for six different specimens, and the average values were recorded.

### Histological Analysis

The samples were fixed by immersion in 4% paraformaldehyde for 48 h and decalcified in 10% EDTA solution for 1 week. Then, the samples were washed repeatedly and gently with normal saline to eliminate the effect of the decalcification solution on tissue staining. The samples were embedded in paraffin and then, serial sections were made perpendicular to the osteotomy surface and stained with the Hematoxylin–Eosin (HE) Staining Kit. After the sections were prepared, a microscope (Olympus, Japan) was used to observe and photograph the sections. ImageJ was used to calculate the depth of the mechanical damage and the thermal damage to the bone. The numbers of bone lacunae with cells and empty bone lacunae within 200 μm of the cutting edge were counted, and the rate of empty bone lacunae was calculated (Yin et al., 2016; Palaia et al., 2020).

### Biological Characteristics of the Osteotomy Surface.

#### Preparation of Bone Slices

**FIGURE 2:**
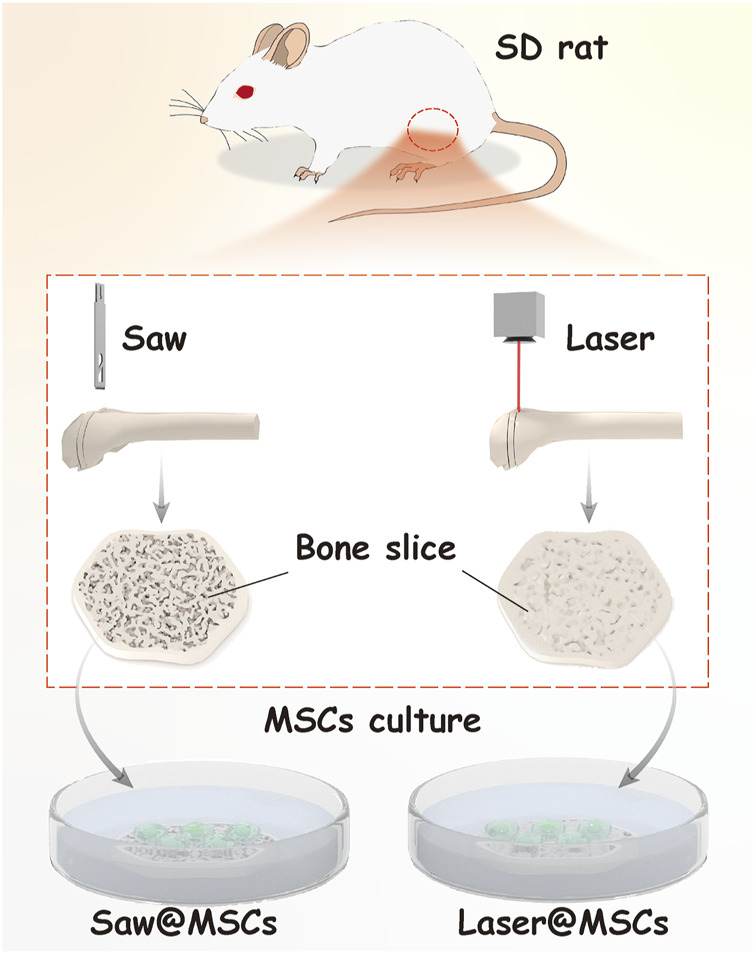
Preparation of bone slices and cell culture.

To observe the biological characteristics of the osteotomy surface prepared by the two cutting methods, a laser and mechanical saw were used to cut the distal femur to prepare the bone slices. Briefly, after the femur specimen was fixed, a laser (the mechanical saw was used in the S group) was used to cut the distal femur into 3-mm-thick slices. Twelve rats were used to prepare bone slices. Since two bone slices could be prepared from one femur, 48 bone slices were prepared and used for all cell experiments. The preparation schematic chart of the bone slice is shown in Figure 2. Then, the samples were soaked in 75% alcohol for 24 h for disinfection.

### Cell Culture

Rat mesenchymal stem cells (MSCs) were isolated from three rats aged 2 weeks (100 ± 10 g) and cultured as previously reported (Mandal and Kundu, 2009). The MSCs were cultured in Dulbecco’s modified Eagle medium (DMEM) supplemented with 10% (v/v) fetal bovine serum (FBS) and 1% penicillin−streptomycin solution at 37 °C in an incubator containing 5% CO_2_, and the culture medium was replaced every second day. The identification of MSC was shown in Supplementary Materials. The MSCs at the 3^rd^ passage were used in all subsequent *in vitro* studies. The cell density of all cell experiments was 2 × 10^4^ cells/well.

### Cell Infiltration

The sterilized samples (six in each group) were placed at the bottom of a 24-well plate and then, the MSCs were inoculated. To allow the cells to settle uniformly on the surface of the bone slice, the cells were suspended during inoculation. The cells were cultured in an incubator containing 5% CO_2_ at 37°C with 100% relative humidity, and the medium was changed every 2 days.

After 2 and 5 days of culture, the culture medium was discarded, and the cells were flushed gently with sterile PBS three times. The cells were stained using the Calcein/PI Live/Dead Cell Viability Kit according to the instructions for use. The fluorescence solution Calcein can make living cells appear green at 494 μm wavelength, and PI can make dead cells appear red at 617 μm wavelength. After staining, a laser confocal scanning microscope (LSCM, LSM900, ZEISS, German) was used to observe and photograph the MSCs adhered to the surface of the bone slices. High-resolution images of Z-stacks were obtained and confocal Z-stacks of the images were processed and reconstructed with ZEN blue edition software (Version 3.3, ZEISS, German). Then, the cells attached to the bone slices were counted, the survival rate was calculated (Lin et al., 2021), and the depth of cell infiltration was measured with the image reconstructed on the *Z*-axis (Zhao et al., 2016).

### Cytoskeleton Staining and Scanning Electron Microscopy

The method of cell inoculation was the same as mentioned previously. There were three samples in each group. After culturing for 48 h, the medium was removed and the samples (half for staining and half for SEM) were flushed with PBS. After that, 4% paraformaldehyde solution and 0.1% Triton X-100 were added sequentially. Finally, the samples were incubated with Rhodamine-phalloidin (1: 500) solution at room temperature for 2 h. After washing with PBS, DAPI was used to stain the nuclei. All samples were imaged *via* the CLSM. The fractal dimension of the cytoskeleton was calculated with ImageJ software. Before SEM, the samples were fixed with 4% paraformaldehyde and subjected to dehydration using graded ethanol, and then the samples were coated with a thin sputtered gold layer.

### Focal Adhesion Staining Analysis

The method of cell inoculation was the same as that of cell infiltration, with three samples in each group. After fixing the samples with the aforementioned method, the cells were incubated with paxillin (rabbit pAb; 1:50) at 4°C overnight. Then, they were incubated with secondary antibodies coupled to Alexa Flour 488 for 1 h and with rhodamine phalloidin (1: 500) solution for 2 h at 37°C. After that, the samples were washed with PBS and the cell nuclei were stained with DAPI. Fluorescence images were obtained using CLSM. Profiles of F-actin (red) and p-paxillin (green) were extracted from the images of CLSM using ImageJ.

### Statistical Analysis

All experiments were performed at least in triplicate. Data are presented as the mean ± standard deviation (SD). Statistical calculations were performed by Student’s *t*-test using SPSS ver.22 software (United States of America). Statistical significance was set to *p < 0.05, **p < 0.01, and ***p < 0.001.

## Results

### Ultra-Pulsed CO_2_ Laser Osteotomy System Complete Animal TKA Bone Cutting

The laser successfully realized the osteotomy of the distal femur of sheep, and no carbonizations were found on the bone. There was a crackling sound during cutting, with the smell of protein burning, and white debris flew out of the incision (Figure 1).

### Laser Osteotomy Preserves the Intact Structure of the Bone Surfaces and Has Better Surface Roughness than Mechanical Saw Osteotomy

**FIGURE 3:**
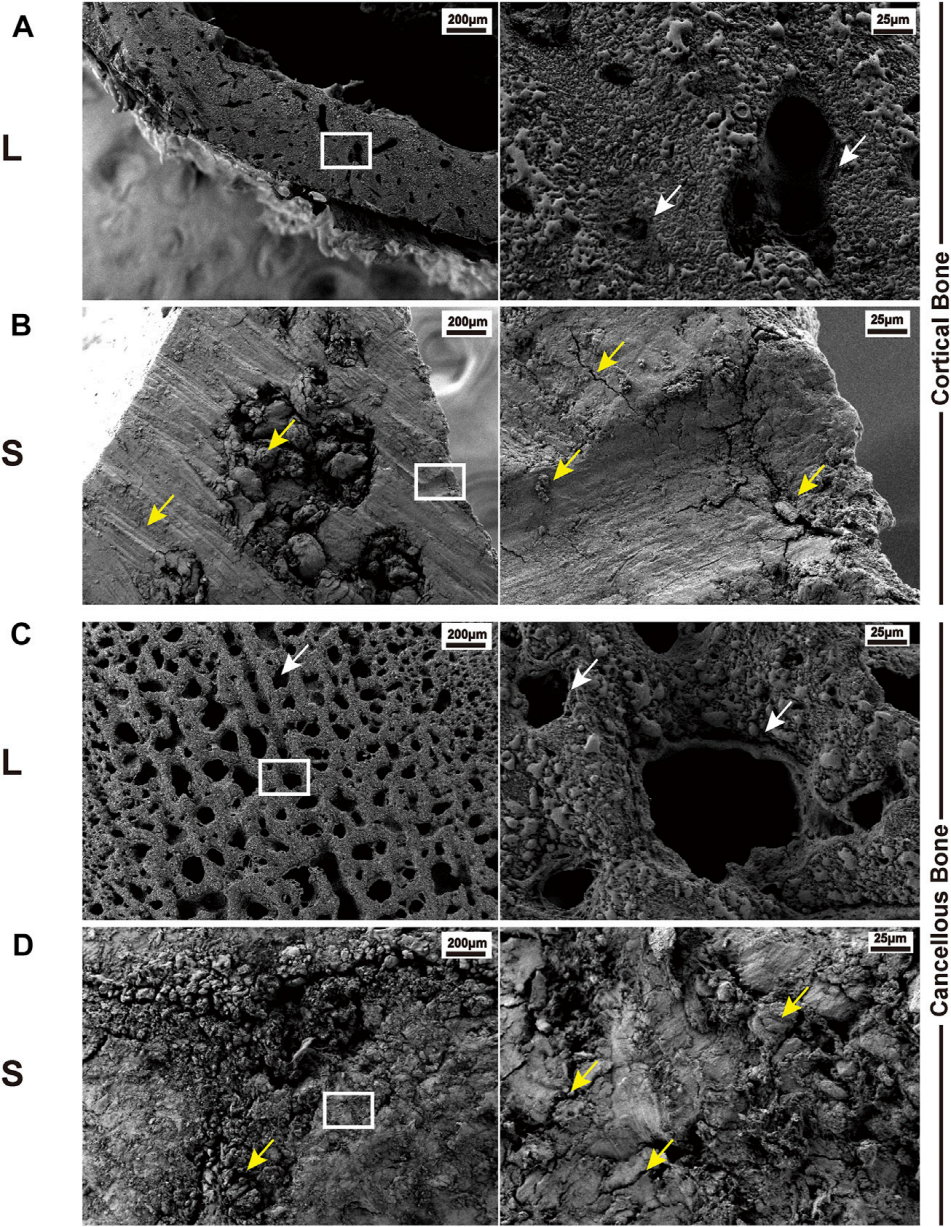
Typical SEM images of bone surface cutting by the laser and mechanical saw. **(A)** Cortical bone surface produced by laser osteotomy; white arrows indicate the trophoblast vascular lumen and Haversian canal. **(B)** Cortical bone surface produced by mechanical saw osteotomy; yellow arrows indicate abrasion marks, cracks, and bone chips caused by the saw blade. **(C)** Cancellous bone surface produced by laser osteotomy; white arrows indicate the trabecular bone and the space between the trabecular bones. **(D)** Cancellous bone surface produced by mechanical saw osteotomy; yellow arrows indicate cracks and bone chips. White boxes indicate the cut-out after the subsequent magnification.

L group: The osteotomy surface retained the original physical structure of the bone, with smooth sections and no mechanical damage. The typical images are shown in Figure 3. An open trophoblast lumen and a Haversian canal were observed on the cortical bone surface (Figure 3A). In addition, on the cancellous bone surface, intact trabeculae and a large number of interlacing pores formed by the trabeculae were observed (Figure 3C).

S group: The surface was severely damaged, showing fragmentary and no porous structure. There were several large cracks on the cortical bone and wear marks caused by saw blade grinding (Figure 3B). Furthermore, the trabeculae could not be distinguished on the cancellous bone surface, and a large number of cracks and bone debris appeared on the surface. The holes between the trabeculae were filled with the bone debris generated by the saw blade (Figure 3D).

**FIGURE 4:**
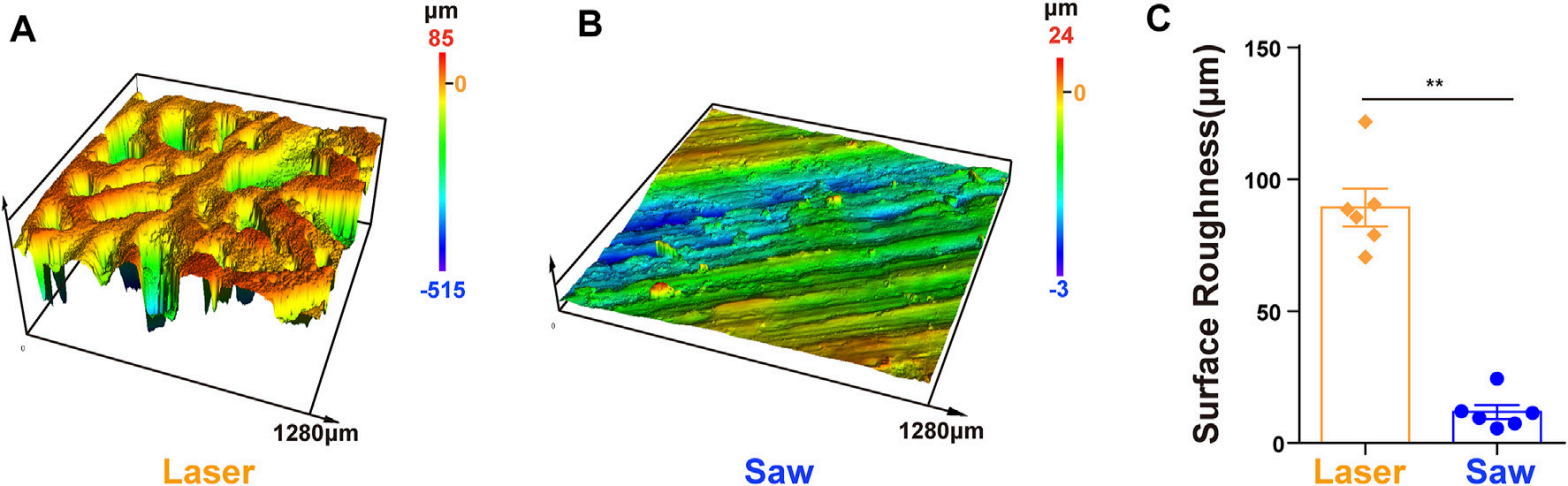
Surface roughness. **(A,B)** 3D height distribution images of the bone surface which were taken with the laser confocal scanning microscope after laser and mechanical saw cutting. **(C)** Surface roughness, **, p < 0.01.

The surface roughness of the L group is significantly higher than that of the S group as shown in Figure 4.

### Laser Osteotomy Does Not Cause Thermal and Mechanical Damage to Bone Tissue and Cells

**FIGURE 5:**
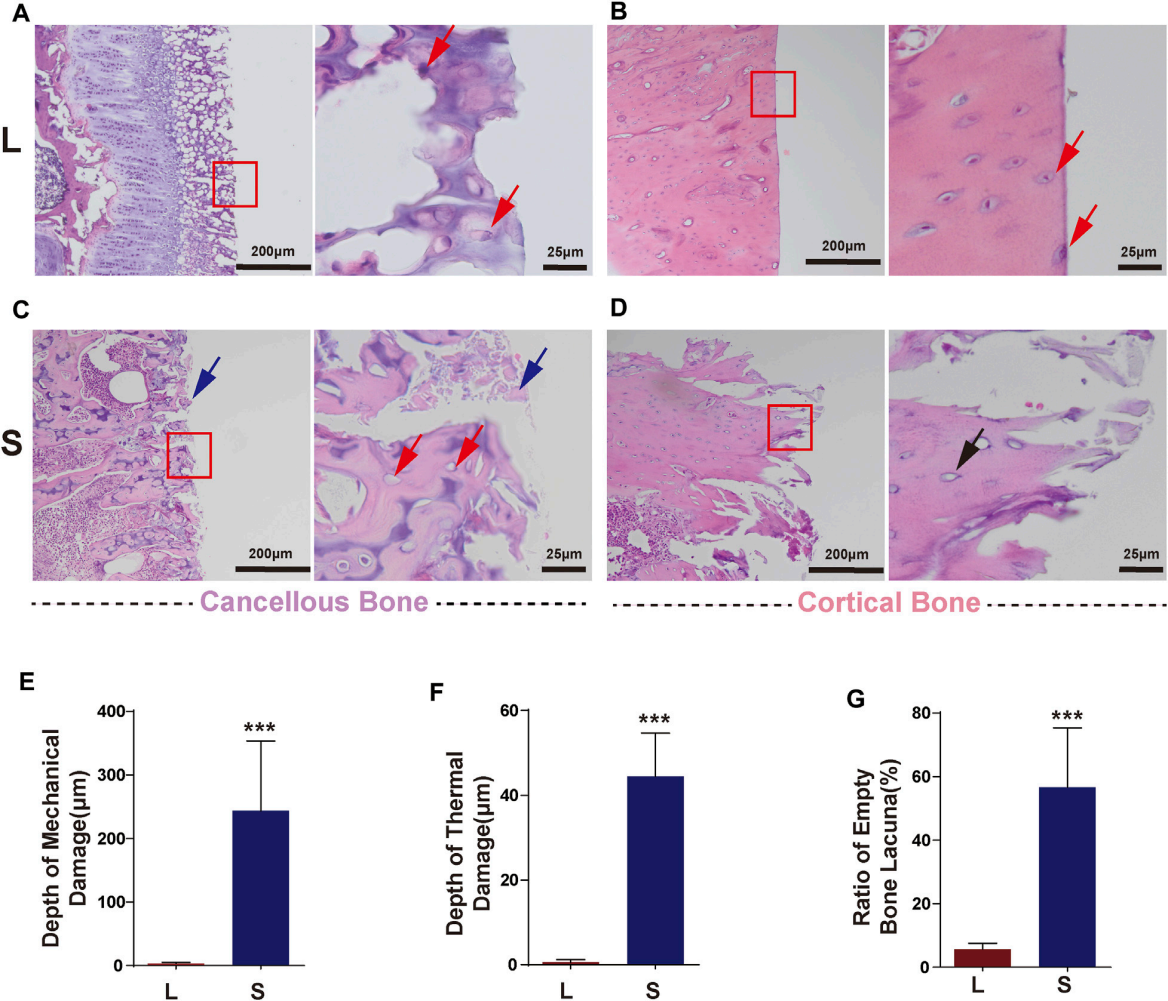
Typical histology images after laser and mechanical saw osteotomy. **(A)** HE images of laser-cut cancellous bone, with intact cells at the cutting edge indicated by red arrows. **(B)** HE images of laser-cut cortical bone with neat cutting edges and intact cells. Red arrows indicate cells on the cutting edge. **(C)** Images of the cancellous bone cut by mechanical sawing, with blue arrows indicating bone chips filled with cutting edges and red arrows indicating empty lacunae and crushed trabeculae. **(D)** HE images of the cortical bone cut by mechanical sawing. Black arrows indicate the destroyed margin bone tissue and empty lacunae. **(E)** Depth of mechanical damage. **(F)** Depth of thermal damage. Boxes indicate cutout after subsequent magnification. **(G)** Empty bone lacunae rate. HE, Hematoxylin–Eosin staining. *** p < 0.001.

L group: The sections were uniformly stained, and it was observed that in the cancellous bone sections, all the bone trabeculae at the cutting edge were intact without thermal or mechanical damage, and the cells remained intact. Typical images are shown in Figure 5. In addition, the cortical bone had a neat cutting edge without mechanical cracks. The cell morphology near the cutting edge was consistent without thermal damage, and there were even intact cells on the cutting edge (Figure 5B).

S group: In the cancellous bone, the bone tissue and cells were seriously damaged, the bone trabeculae were fragmented, and the pores between the bone trabeculae were filled with a large amount of bone debris (Figure 5C). A large number of empty bone lacunae left after the cells cracked and disappeared. The damage also occurred in the cortical bone with more pronounced thermal damage (Figure 5D). In the S group, the mechanical damage depth was 243 ± 44 μm (Figure 5E), the thermal damage depth was 44 ± 10 μm (Figure 5F), and the empty bone lacunae rate was 55.6 ± 7% (Figure 5G), which were significantly higher than those in the L group.

### Osteotomy Surface Biological Studies: Infiltration, Cytoskeleton, and Focal Adhesion Analysis

**FIGURE 6:**
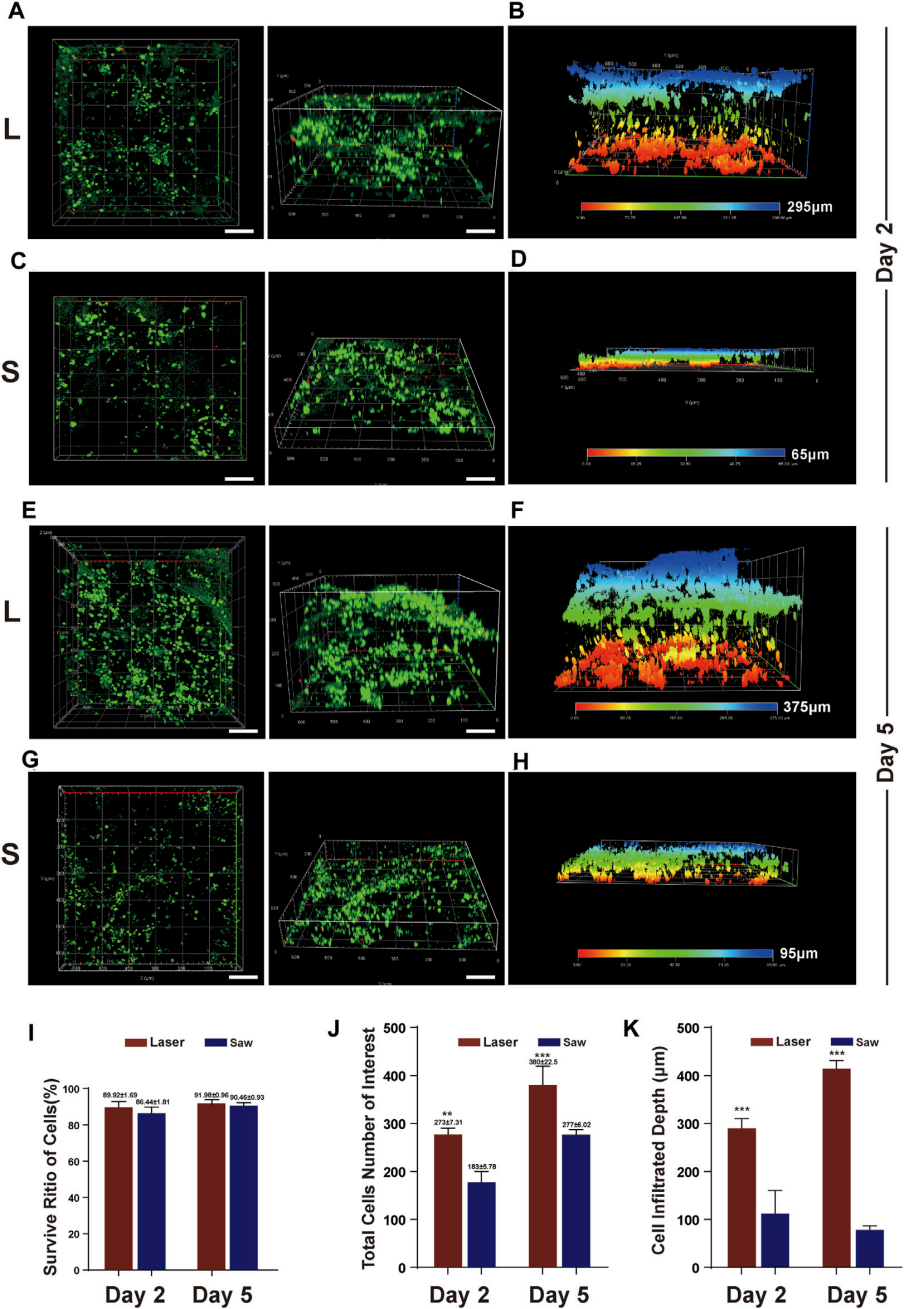
MSC infiltration into bone surface. **(A)** Typical images of z-axis reconstruction of adhesive MSCs on bone slices at Day 2 after the cell inoculation (culture) in L group, **(B)** infiltration depth at Day 2 after the cell inoculation in L group. **(C)** Typical images of z-axis reconstruction of adhesive MSCs on bone slices at Day 2 after the cell inoculation in S group, **(D)** infiltration depth at Day 2 after the cell inoculation in S group. **(E&G)** Typical images at Day 5 after the cell inoculation in the two groups. live = green, red =dead and scale bar = 100 μm. **(F&H)** infiltration depth at Day 2 after the cell inoculation in the two groups. **(I)** Quantification of viable cell percentage in five scopes in L and S group. **(J)** Quantification of total number of MSCs in five scopes in L and S group. **(K)** Quantification of MSCs infiltration depth in five scopes in L and S group. **, p < 0.01, ***, p < 0.001.

MSCs were evenly distributed on the surface of the samples. It was observed that more MSCs adhered in the L group than in the S group on day 2. The same results were also observed on day 5, and the differences were statistically significant (*p* < 0.001). More importantly, in the L group, the infiltration depth of the MSCs was greater than that in the S group, and the difference was statistically significant (*p* < 0.001). There was no difference in the survival rate of the MSCs between the two groups. Typical images are shown in Figure 6.

**FIGURE 7:**
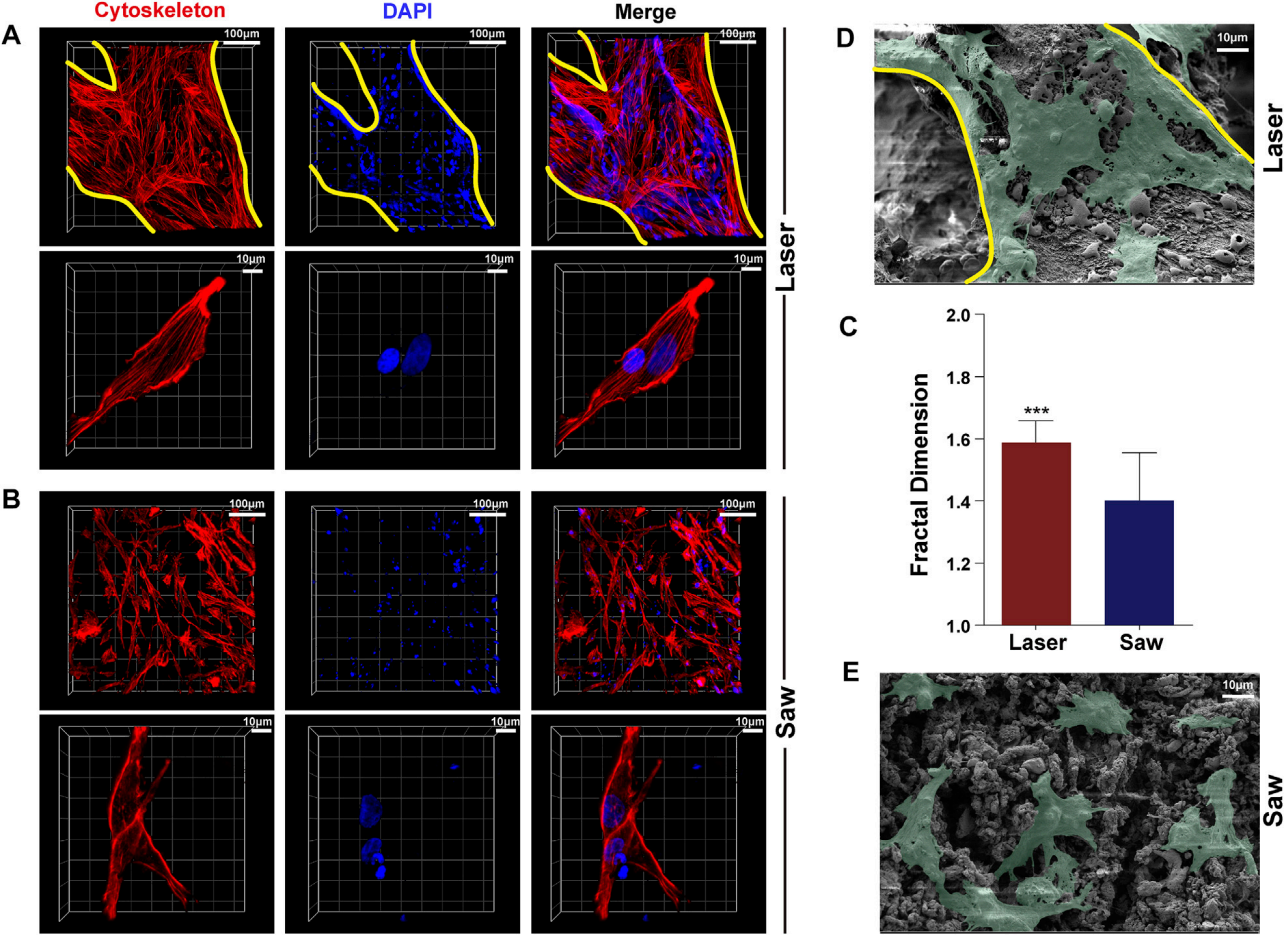
Typical images of adhesion of MSCs. **(A)** is the laser group, **(B)** is the mechanical saw group, photographed by using a laser confocal scanning microscope, and red represents the cytoskeleton, blue represents the nucleus, and the yellow line represents the trabecular boundary. **(C)** Statistics of the fractal dimension of a single MSC cytoskeleton of the two groups (*n* = 50) **(D,E)** are scanning electron microscopy images of MSC adhesion to the bone surface of the two groups (green).

A typical comparison of MSC cytoskeleton staining images in the L and S groups is shown in Figure 7. It shows that the cells were well spread out in the L group, having a tendency to extend along the trabecular direction, and the cytoskeleton polarization was obvious. However, in the S group, the cells were randomly adhered on the bone surface, and the cytoskeleton polarization appeared only at the cell edge. Moreover, the fractal dimension of the cells in the L group is better than that in the S group. The SEM images further indicate that the cells in the L group were more spread out than those in the S group.

**FIGURE 8:**
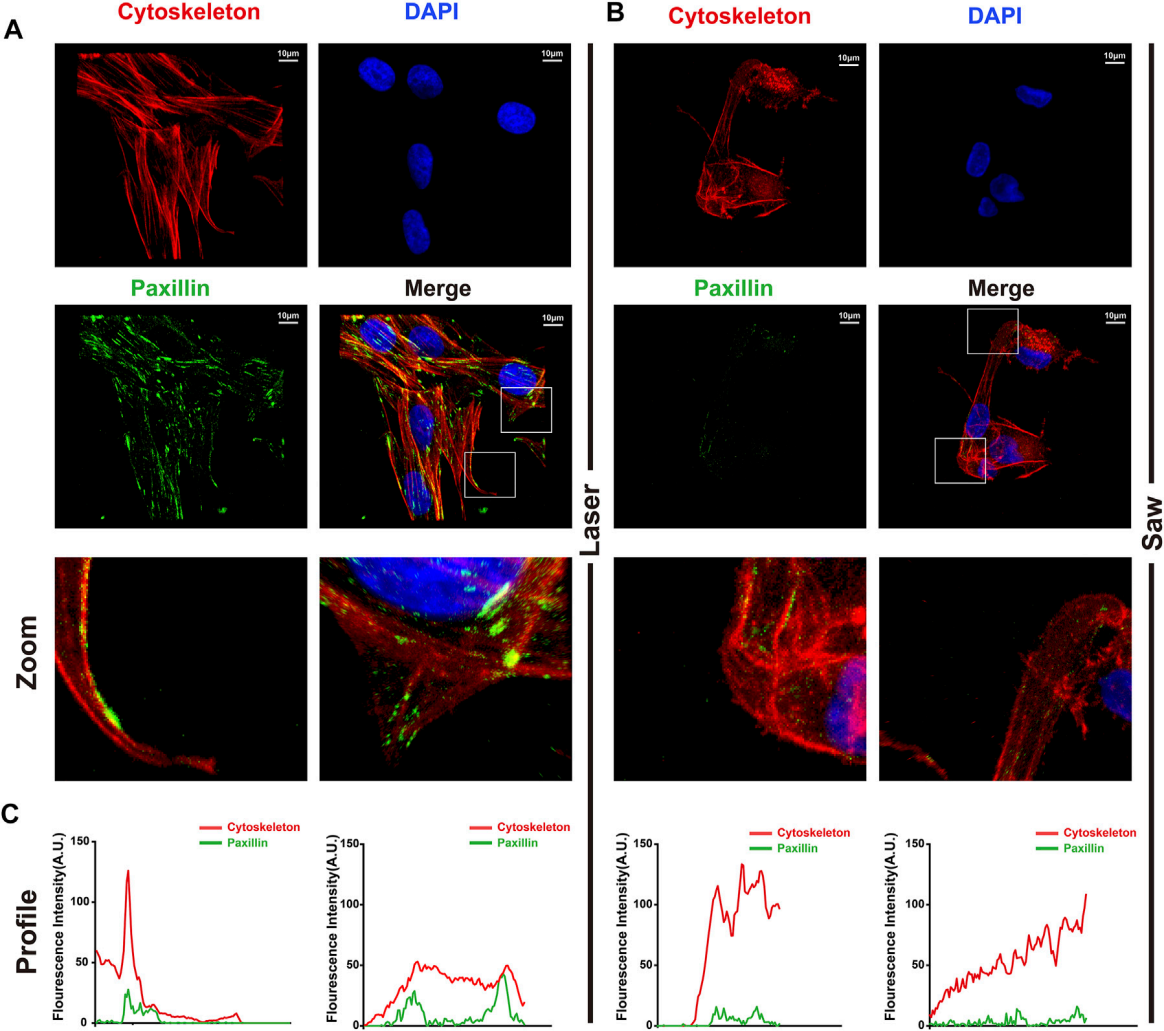
Typical images of laser cutting bone surface promoting the formation of focal adhesion. **(A)** The laser group and **(B)** the mechanical saw group were photographed by laser confocal microscope. The red is the cytoskeleton, the green is the paxillin, and the blue is the nucleus. **(C)** Profiles of F-actin (red) and p-paxilin (green) were extracted from images (white segments) using ImageJ software.

To better understand how cell adhesion was altered in the two groups, MSCs were immunostained with F-actin and paxillin to examine FA dynamics. The paxillin and the cytoskeleton co-located showed focal adhesion. Typical images are shown in Figure 8. MSCs in the L group developed numerous, larger FAs. In contrast, we noted that the S group only exhibited a dot-like staining pattern near the cell periphery.

## Discussion

In this study, we used an improved ultra-pulsed CO_2_ laser osteotomy system to successfully achieve the five facial and box osteotomy of TKA in sheep knees, and no thermal damage such as carbonization occurred during the experiment (Figure 1C). At present, laser has been successfully applied in medical treatment, such as in ophthalmology and oral surgery (Sugar and Alan, 2002; Liu et al., 2006; Zhang et al., 2011). Theoretically, lasers are the most promising alternative to conventional mechanical tools for performing accurate osteotomy. Furthermore, laser has the advantages of non-contact, non-vibration (Berrocal et al., 2007), high-precision, and arbitrary-geometric-shape cutting (Baek et al., 2015; Marcello et al., 2018). Moreover, improved laser cutting methods and the newly developed laser can perform osteotomy with controlled thermal impact. Ivanenko et al. (2005) and Kuttenberger et al. (2010) successfully performed pulsed CO_2_ laser osteotomy without thermal damage to animals. However, so far, there are few reports describing the characteristics of the bone surface after laser cutting. The bone surface is very important, especially in operations that require complete contact between the bone surface and the implant to achieve bone integration, such as TKA. Therefore, we further analyzed the bone surface structure and biological characteristics after laser cutting and compared them with traditional mechanical osteotomy tools.

To better protect animals and conduct subsequent cytological experiments, the animal model was adjusted to SD rats. The results of scanning electron microscopy (SEM) on the osteotomy surface of rat femur showed that mechanical sawing could seriously damage the bone structure, whereas laser could completely preserve the topological structure of the bone. Mechanical saw cutting led to severe damage of the bone trabeculae, producing a large amount of bone debris filling the pores and resulting in the complete destruction of the bone trabecula structure. In addition, many cracks and abrasion marks after sawing appeared in the cortical bone. Studies have shown that bone debris will increase aseptic inflammatory response, resulting in prolonged bone healing time (Giraud et al., 1991). Such results may have an adverse effect when cementless prostheses are used. On the contrary, laser can cut into a completely different bone surface. In the cancellous bone, the trabecular bone on the osteotomy surface remains intact with a large number of pores in it. At the same time, the cortical bone in the middle part of the femur has a smooth bone surface and many complete open Haversian canals. The surface roughness analysis shows that the laser-cut surface has better performance in roughness. Combined with the SEM images, we believe that laser can achieve ideal osteotomy while maintaining the complete natural topological structure of the bone, which may be conducive to the early stabilization of cementless prostheses and the realization of long-term bone integration.

Previous studies have shown that a micromotion larger than 150 μm can inhibit bone ingrowth onto porous surfaces and consequently lead to implant failure (Justy, 1997). While it is difficult to achieve the desired bone cutting with traditional mechanical cutting tools, Macdonald et al. (2004) reported an error of more than 2 mm when using traditional mechanical cutting tools during TKA bone preparation. Apparently, laser cutting has no such concerns. The laser can greatly reduce the incision width to 100 μm. The specific width depends on the focal spot diameter. In this study, the focal spot diameter is 60 μm. In addition, the cutting geometry is not restricted and arbitrary and complex cutting shapes in the geometry are available. Of course, laser must be combined with computer and robot tools to achieve accurate osteotomy (Burgner et al., 2010).

Significantly, the laser osteotomy system used in this study can further reduce thermal damage to the bone. By performing H–E staining, we found that intact cells were preserved at the cutting edge of laser-cut bone tissue. In contrast, mechanical sawing resulted in severe mechanical damage to bone tissue (cracks up to 500 μm deep) and thermal damage (incomplete damage to cells within 200 μm of the cutting edge). The previous research on CO_2_ laser osteotomy has ever been plagued by thermal damage. In the early studies, a continuous CO_2_ laser was used for bone cutting experiments, which resulted in severe carbonization of bone tissues (Panossian et al., 1984). Later, Frentzen et al. (2003) used a pulsed CO_2_ laser to cut pig ribs with a water jet. They reported thermal damage to the bone tissue only within10 μm near the cutting edge and incomplete damage to bone tissue cells within 50 μm of the edge.

We can analyze these results by the following mechanisms of action of laser osteotomy. 1) First, laser parameters determine the effect of laser irradiation on the tissues ^[34]^. When laser energy reaches the surface of the bone, it can be reflected, scattered, absorbed, or transmitted to the surrounding tissues. The 9.3-μm wavelength laser we used has high absorption in bone tissue. Since bones mainly comprise water and inorganic calcium salts, a rapid phase transformation from water to gas will result in the micro-rupture and micro-explosive removal of bone mineral phases (Gunaratne et al., 2016). However, the removal only occurred in the bone tissue irradiated by the laser (the focus diameter of the beam in this study was 60 microns), so there was little mechanical damage to the nearby bone tissue. 2) The second mechanism is that the laser can transmit the required energy in a short time (rise time 10 µs, fall time 15 µs, and pulse duration 25 µs), and the energy has little time to diffuse into the surrounding tissues. 3) At the same time, water acts as a coolant to reduce thermal injury (Zhang et al., 2012), so almost no heat damage was found in the tissues, while mechanical tools, cutting with strong mechanical forces, will inevitably damage nearby bone tissues and the repeated grinding of the saw blade will also raise the temperature and cause tissue and cell damage.

Cell adhesion is an important factor affecting the ability of bone integration (Zhao et al., 2013; Taale et al., 2018; Kelly et al., 2021). Therefore, we further studied the adhesion of MSCs to the bone surface prepared by the two cutting tools. The results showed that both the number of cells adhered to the bone surface, and the infiltration depth in the laser group was greater than that in the control group. The cells adhered to the bone surface prepared by laser reside in a comfortable environment, while the cells adhered to the bone surface cut by the mechanical saw seem to be damaged. Judging from those results, we believe that laser cutting retains the original topological structure of the bone surface, which is more conducive to the adhesion of MSCs. In addition, the porous bone surface obtained by laser cutting means that the surface area of the bone surface is increased. The results may be beneficial for bone integration.

### Limitations

There are also limitations in this study, and further research is still needed. First, the width of the incision for laser osteotomy is 1 mm, which greatly reduces the efficiency of laser cutting. The reason is that the optical transmission design is insufficient and the optical path design needs to be further optimized. Second, the average power of the laser used in the system is low, which limits the speed of laser cutting.

## Conclusion

In this study, we realized the designed laser TKA osteotomy of animal knee joints and found that the laser-cut bone surface can retain the natural topology of the bone, which is conducive to cell adhesion and infiltration and may promote the bone integration of prosthesis.

## Data Availability

The raw data supporting the conclusion of this article will be made available by the authors, without undue reservation.
