# Supplementary material for: Ultra-Pulsed CO2 Laser Osteotomy: A New Method for the Bone Preparation of Total Knee Arthroplasty
Source: Front Bioeng Biotechnol. 2022 Apr 29;10:858862. doi: 10.3389/fbioe.2022.858862 (PMC9096707; doi:10.3389/fbioe.2022.858862)
Supplement: Supplementary file 1 [file DataSheet1.DOCX]

1. **Identification of BMSCs:**

The phenotype of P3 generation were identified by flow cytometry. When the fusion degree of P3 generation cells were close to 80%, the culture medium was removed, then the cells were gently moistened with PBS solution, and then the adherent cells were digested with 0.25% trypsin. The cells were centrifuged for 5 minutes at the speed of 1000 rpm/min at 4 ℃. After PBS moistened the cells twice, the self-made flow buffer was added to adjust the cell density to 2 × 10^6^/mL. 1.5mL EP tubes were selected and labeled with primary antibody. 200μL cell suspension was added to each tube, and then 2μL primary antibody was added to each tube. The tubes were shaken evenly and incubated at 4℃ for 30 min. At the end of incubation, 1 mL flow buffer was added to each tube and mixed, centrifuged at 1000 rpm/min for 5 min, then supernatant was discarded and cells were washed twice. Flow buffer was added at 200 μL/ tube, and then 2μL fluorescence secondary antibody was added to resuscitate the cells. All EP tubes were placed at 4℃ and incubated for 30 min under dark conditions. After incubation, 1 mL flow buffer was added to each tube for washing twice, and then 400 μl flow buffer was added. After suspension, the test results were analyzed by F1owJo 7.6 software. Flow cytometry antibodies are shown in Table 1

Table 1 Flow cytometry antibody list

| Primary antibody | Secondary antibody |
| --- | --- |
| Anti-rat CD90 | PE-Goat anti-mouse IgG |
| Anti-rat CD73 | PE-Goat anti-mouse IgG |
| Anti-rat CD34 | PE-Goat anti-mouse IgG |
| Anti-rat CD45 | PE-Goat anti-mouse IgG |

**2 Morphology of BMSCs**

Microscopic observation showed that the primary cells grew like colonies and showed a typical swirl-like arrangement, as shown in Figure 1. Individual cells showed a fusiform morphology, showing fibroid cell characteristics.


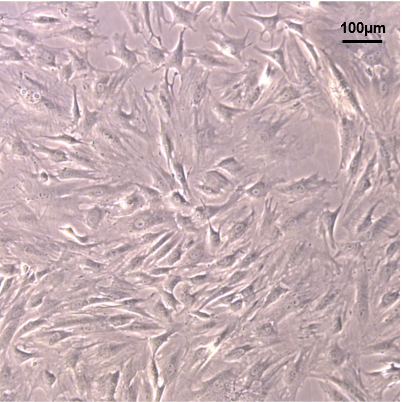


**Figure 1** Morphological properties of BMSCs (Scale bar = 100 μm)

**3 Expression of BMSCs surface markers**

As shown in Figure 2, the results of flow cytometry identification showed that surface markers CD90 and CD73 for the isolated and purified BMSCs were expressed with positive rates of over 90%, while almost no hematopoietic stem cell surface markers such as CD45 and CD34 were expressed (positive rates were less than 0.1%). The results showed that the cells we isolated were in line with the surface antigen marker characteristics of BMSCs, indicating that the BMSCs we isolated and cultured could meet the requirements of the experiment.


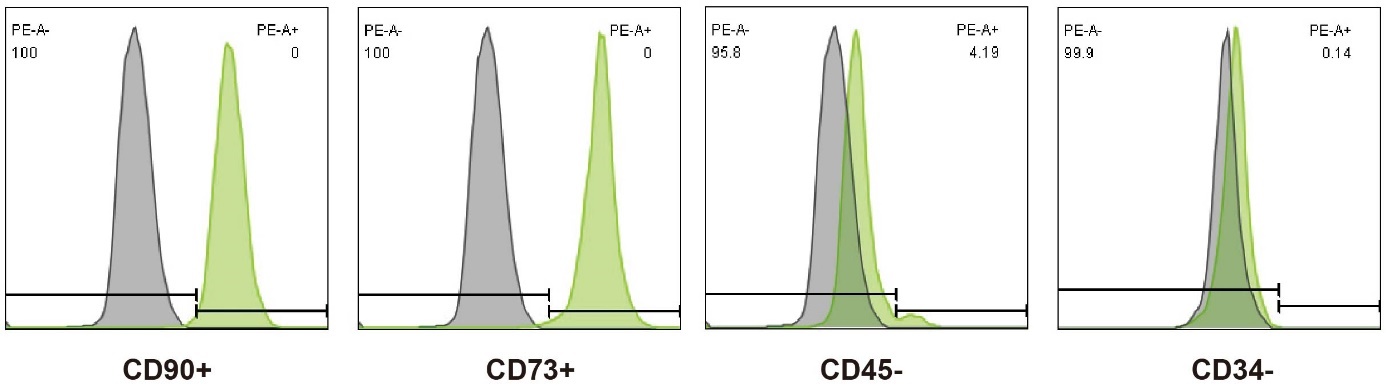


**Figure 2** Surface markers of BMSCs
